# Benefits of Elective Para-Aortic Radiotherapy for pN1 Prostate Cancer Using Arc Therapy (Intensity-Modulated or Volumetric Modulated Arc Therapy): Protocol for a Nonrandomized Phase II Trial

**DOI:** 10.2196/11256

**Published:** 2018-12-13

**Authors:** Cédric Draulans, Steven Joniau, Valérie Fonteyne, Louke Delrue, Karel Decaestecker, Wouter Everaerts, Piet Dirix, Laura Van den Bergh, Wouter Crijns, Hans Vandendriessche, Lodewijk Van Wynsberge, Piet Ost, Nicolaas Lumen, Pieterjan Buelens, Karin Haustermans, Charlien Berghen, Gert De Meerleer

**Affiliations:** 1 Department of Radiation Oncology University Hospitals Leuven University of Leuven Leuven Belgium; 2 Department of Urology University Hospitals Leuven University of Leuven Leuven Belgium; 3 Department of Radiation Oncology Ghent University Hospital University of Ghent Ghent Belgium; 4 Department of Radiology Ghent University Hospital University of Ghent Ghent Belgium; 5 Department of Urology Ghent University Hospital University of Ghent Ghent Belgium; 6 Department of Radiation Oncology Iridium Cancer Network Wilrijk, Antwerp Belgium; 7 Department of Radiation Oncology Limburg Oncology Centre Hasselt Belgium; 8 Department of Urology AZ Jan Portaels Vilvoorde Belgium; 9 Department of Urology General Regional Hospital Tienen Tienen Belgium

**Keywords:** elective para-aortic radiotherapy, external beam radiotherapy, PART trial, prostate cancer

## Abstract

**Background:**

In patients with prostate cancer (PCa) with histopathologically proven pelvic lymph node (LN) metastasis (pN1) after extended pelvic lymph node dissection (ePLND), multimodality treatment consisting of treatment of the primary tumor and whole pelvic radiotherapy (WPRT) combined with androgen deprivation therapy (ADT) offers promising results, leading to better cause-specific survival rates compared with ADT alone. However, in case more than one pelvic LN is invaded by the tumor, approximately 40% of the patients relapse biochemically and clinically. Clinical relapse is present in the para-aortic LNs (M1a disease) in up to 77% of the relapsing cases.

**Objective:**

We hypothesize that, based on the evidence that positive LNs represent the door to hematogenous dissemination, elective para-aortic irradiation will reduce the development of both retroperitoneal nodal (M1a) and distant metastasis (M1b or M1c disease), postpone the need for palliative ADT, and prolong the time to castration-refractory disease.

**Methods:**

To test this hypothesis, we will conduct a prospective, nonrandomized phase II trial to study the efficacy of additional elective para-aortic radiotherapy (PART) in pN1 patients compared with those who were historically treated with adjuvant WPRT alone. We aim to include 137 patients with PCa and presence of pN1 disease after ePLND. With this number of patients, an improvement of 15% in the 5-year clinical relapse-free survival can be detected with a power of 80%.

**Results:**

Recruitment of patients for this trial started in 2017 and will be completed approximately by March 2020.

**Conclusions:**

This is the first phase II trial to investigate the benefits of an elective PART in patients with PCa. The results of this trial will potentially serve as a sound base for a later randomized phase III trial. All participants are given a PART information sheet and required to give written informed consent. Results are expected to be published in a peer-reviewed journal.

**Trial Registration:**

ClinicalTrials.gov NCT03079323; https://clinicaltrials.gov/ct2/show/NCT03079323 (Archived by WebCite at http://www.webcitation.org/73ELimv1d)

**International Registered Report Identifier (IRRID):**

PRR1-10.2196/11256

## Introduction

Prostate cancer (PCa) is the most common nonskin malignancy and an important cause of cancer-related mortality in men in industrialized countries worldwide [1,2]. Mortality is highest in patients with high-risk PCa, defined by the guidelines of the European Association of Urology (EAU) as T-stage ≥cT2c or Gleason score ≥8 or prostate-specific antigen (PSA) >20 ng/mL. These patients benefit from aggressive local treatment (surgery with or without adjuvant radiotherapy or primary radiotherapy). To assess the risk of disease spread to pelvic nodes, predictive nomograms are used [3-5], although the EAU guidelines consider an extended pelvic lymph node dissection (ePLND) as a necessity in high-risk patients [6]. Indeed, ePLND has proven to be the most accurate nodal staging procedure and, therefore, remains the gold standard [7] with even a positive effect on PCa mortality, certainly in case of limited nodal disease [8] and negative nodes [9].

Historically, patients with positive pelvic lymph nodes (LNs; N1) were considered metastatic and treated with lifelong palliative androgen deprivation therapy (ADT) only [10]. However, in the 21st century, an important paradigm shift occurred. First, local treatment with curative intent is gaining interest in patients with N1 disease [11]. Hereby, also the extent of ePLND plays a crucial role in predicting cause-specific survival (CSS) as demonstrated by Abdollah et al [12]. Second, large retrospective series demonstrated an improvement in prostate cancer-specific survival (PCSS) when postoperative radiotherapy was added to ADT in pathologically node-positive (pN1) patients [13-15].

In the multidisciplinary approach for pN1 patients, multimodality treatment consisting of treatment of the primary tumor, long-term ADT, and whole pelvic radiotherapy (WPRT) has become the standard of care at the Leuven University Hospitals (LUH; Leuven, Belgium) and at the Ghent University Hospital (GUH; Ghent, Belgium). WPRT is delivered using intensity-modulated or volumetric modulated arc therapy (IMAT/VMAT) [16,17]. Clinical results demonstrated that this multimodality treatment is well tolerated and results in 5-year PCSS of >90%, with the best results observed in patients having a low number of positive LNs. Indeed, patients presenting with 1 or 2 positive LNs had a 5-year PCSS comparable to that of pN0 patients [18-20].

The number of pathologically metastatic LNs is a determinant for patient outcome. In case >2 LNs are pathologically invaded by the tumor, 30%-40% of the patients relapse biochemically and clinically [21,22]. Furthermore, some data suggest that extracapsular extension of pelvic nodal metastases is an important negative prognostic factor in pN1 patients [23].

**Figure 1:**
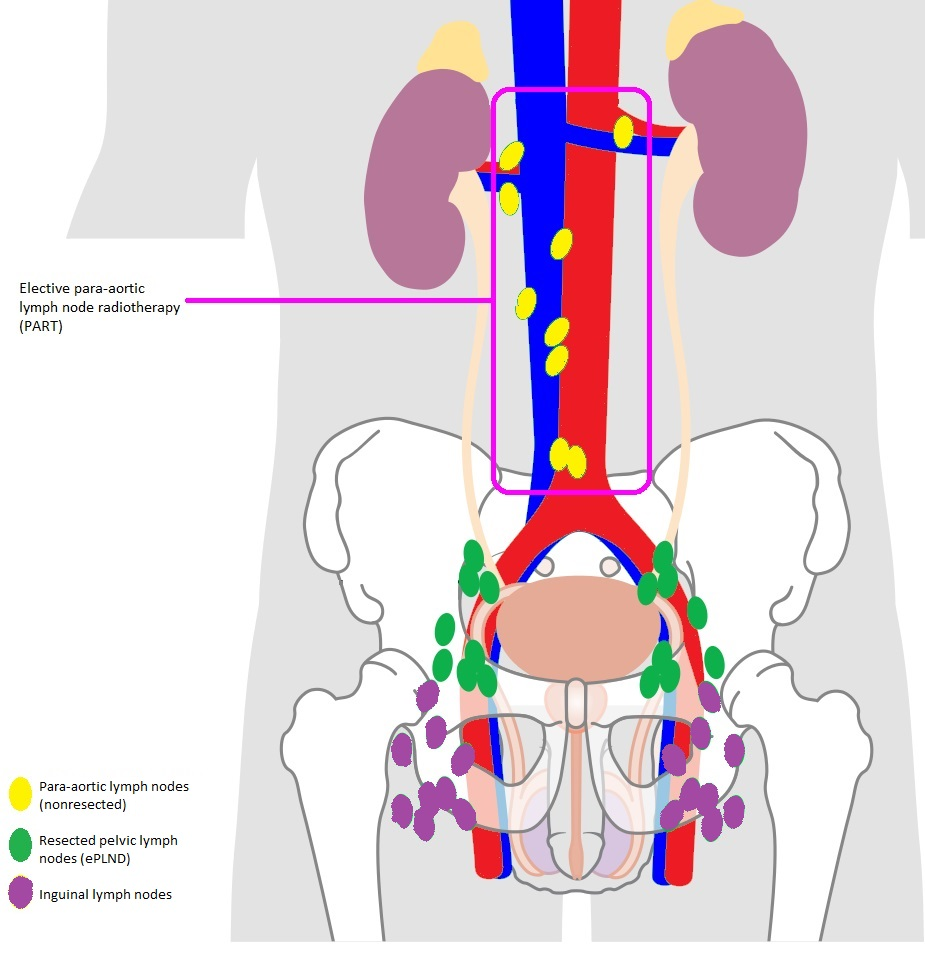
Graphic presentation of the study hypothesis.

Reportedly, clinical relapse (cR) is present in the para-aortic LNs (PALN, M1a disease) in up to 77% of the cases [24,25]. Rischke et al demonstrated the retroperitoneum to be the most frequent site of relapse after pelvic salvage treatment [26]. In the TNM classification, patients with positive PALN are denominated M1a disease and considered as a separate entity [27]. We hypothesize that these positive PALNs will lead to further hematogenous spread (M1b-M1c disease [27]) and that elective para-aortic irradiation will decrease the rate of further metastatic spread, postpone the need for palliative ADT, and prolong the time to castration-refractory disease. To test this hypothesis, we designed a prospective, nonrandomized phase II trial to evaluate the efficacy of elective para-aortic radiotherapy (PART) in pN1 patients compared with those who were historically treated with adjuvant WPRT alone (Figure 1).

This trial is novel and unique as it is the first to investigate the irradiation of the PALN region in pN1 patients with PCa to prevent further metastasis. This strategy has already been evaluated in advanced cervical cancer, but to the best of our knowledge, never in patients with PCa.

## Methods

### Study Design

The PART trial is a nonrandomized phase II trial that was approved by the Medical Ethical Committee of the LUH (EC number: B 3222 0163 0604) and is written in accordance with the Standard Protocol Items: Recommendations for Interventional Trials guidelines. Patients are recruited during the multidisciplinary consultation Urology-Radiation Oncology and at the Department of Radiation Oncology of the LUH, GUH, and other participating centers. After giving informed consent, they are included in the trial.

### Inclusion and Exclusion Criteria

Men aged >18 years with histologically proven adenocarcinoma of the prostate at biopsy (cT1-4) who are referred for primary high-dose radiotherapy or patients after radical prostatectomy (RP; pT2-4) with presence of pN1 disease after ePLND are eligible for the study. pN1 disease is defined as the presence of regional LN metastasis in the true pelvis. These regions include the common iliac nodes, presacral nodes, external and internal iliac nodes, and obturator nodes [27]. In all participating centers, performing an ePLND is the standard of care in high- intermediate and high-risk patients, independent of whether the primary treatment is RP or high-dose radiotherapy [7,28]. If pN1 disease is present, patients are eligible if one of the following criteria is fulfilled:

1. Two or more positive LNs
2. Positive LNs/removed LNs >7%
3. Presence of extracapsular metastatic extension at the level of any LN

ePLND is defined as the removal of LNs around the external and internal iliac vessels and in the obturator fossa. Removal of additional LNs in the presacral area or around the common iliac vessels is at the discretion of the treating physician (but strongly advised if present on preoperative imaging). The minimum harvest of removed LNs that is considered representative is set at 14. Textbox 1 summarizes the inclusion and exclusion criteria.

After amending, patients with pN1 disease in the salvage setting will be allowed in the PART trial and will be prospectively followed with the same treatment protocol and study design to obtain data about acute and late toxicity. These data will be analyzed separately and will not interfere with the initial set-up of statistics.

### Radiotherapy: Structure Delineation, Planning and Delivery

#### Structure Delineation

Details on delineation of the clinical target volume (CTV) of the pelvic nodal areas are provided elsewhere [29]. Briefly, the elective LN areas consisted of the obturator, internal and external iliac, presacral, and common iliac nodes [16]. Concerning the prostate bed (postoperative setting) and the prostate (primary setting), both T2-weighted magnetic resonance imaging (MRI) and computed tomography (CT) images are used to optimize delineation, as detailed elsewhere [30-32].

Delineation of the PALN starts caudally at the level where the abdominal aorta splits into both common iliac branches and stops cranially at the level of the renal artery and vein. The CTV of the PALN is created by adding a 7-mm 3-dimensional expansion to the abdominal aorta and inferior caval vein, excluding intestinal loops and vertebral bodies. Unless kidney function is impaired, CT imaging is done using intravenous contrast to optimize the visualization of the vessels and improve discrimination with the intestinal loops. The use of oral contrast to better visualize these intestinal loops is left at the discretion of the treating physician. Details concerning protocols on bladder filling and rectal preparation are provided elsewhere [33]. The planned target volume (PTV) of the LNs is created by expanding the CTV with an isotropic margin of 7 mm.

**Figure 2:**
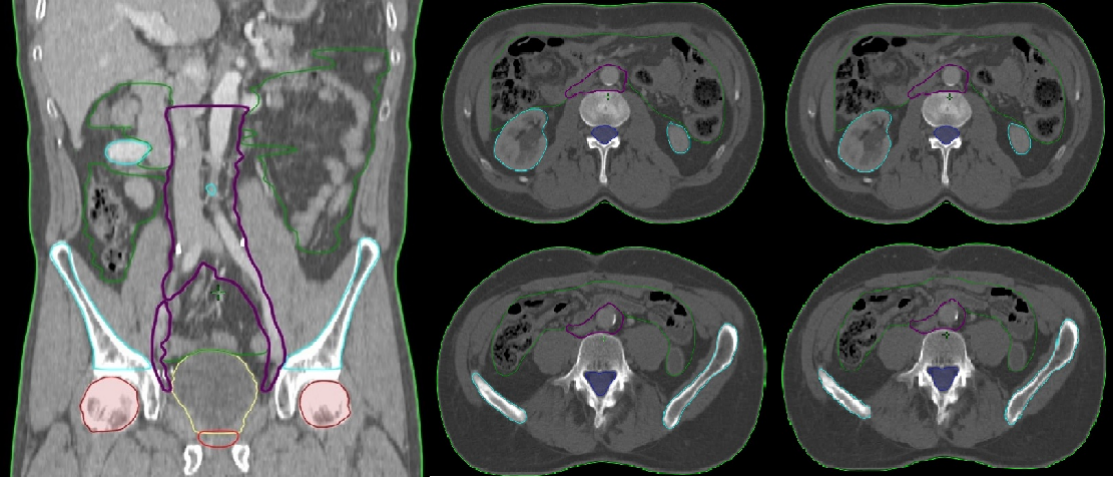
Graphic presentation of the delineation: clinical target volume (CTV)-para-aortic lymph node (LN)+CTV pelvic LN: purple; CTV prostate bed: red; bladder: yellow; sigmoid colon, small intestine, and large bowel: green; femoral heads: brown; bone marrow: light blue; kidneys: turquoise; spinal cord and cauda equina: marine blue.

Concerning the organs at risk (OARs), the following structures are delineated: bladder, anal canal, rectum, sigmoid colon, small intestine, large bowel, femoral heads, spinal cord, cauda equine, bone marrow, and kidneys. Figure 2 depicts the delineation of the OARs.

#### Radiotherapy Planning

**Figure 3:**
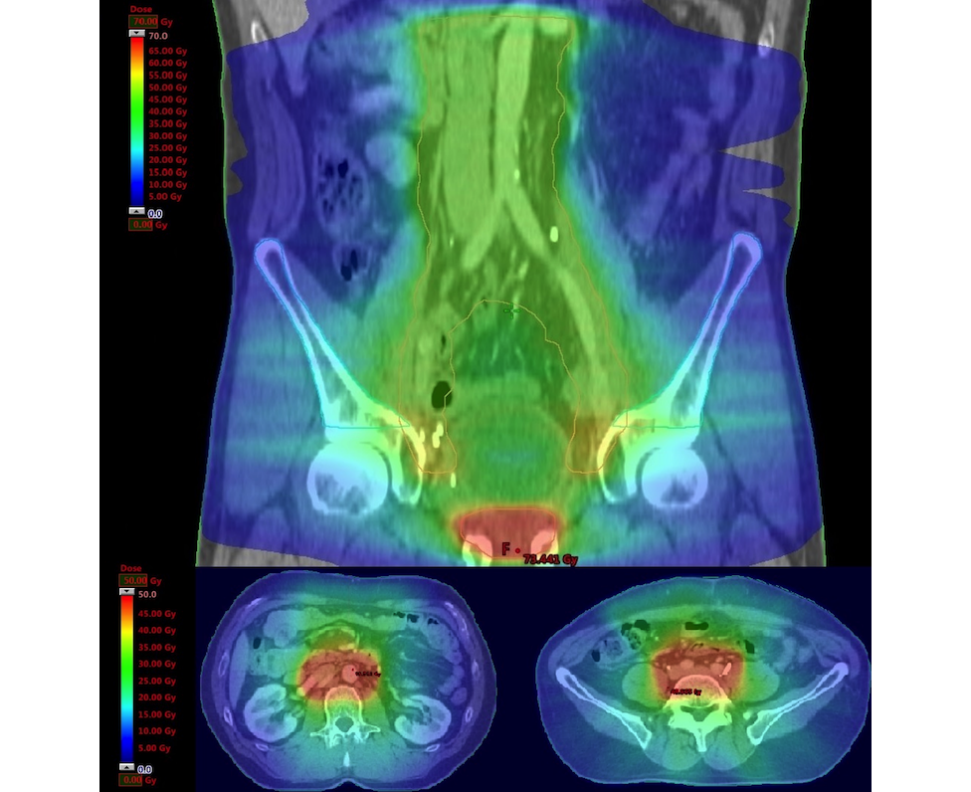
Dose distribution in para-aortic radiotherapy trial. Top: coronal dose distribution (dose range: 0-70 Gy); bottom: transverse dose distribution clinical target volume-para-aortic lymph node (dose range, 0-50 Gy).

The applied planning technology is IMAT/VMAT/RapidArc (Varian Medical Systems, Palo Alto, CA, USA; Elekta, Stockholm, Sweden; Figure 3) [17]. The technology and feasibility to treat the PALN have been published [34].

**Para-aortic radiotherapy (PART) trial: inclusion and exclusion criteria.**

**Inclusion criteria**

- Signed informed consent and willingness to comply with the treatment and follow-up
- Diagnosis of histopathologically confirmed prostate cancer
- No former treatment for prostate cancer, except radical prostatectomy and extended pelvic lymph node dissection (ePLND)
- Presence of pathologically node-positive (pN1) disease after ePLND (criteria of pN1 disease defined in the protocol)
- Age >18 years
- Karnofsky Performance score >70
- Ability to understand the informed consent (Helsinki Declaration)

**Exclusion criteria**

- Recurrent disease status defined as rising prostate-specific antigen after nadir postoperatively
- Presence of cM1a, cM1b, or cM1c disease [27]; patients with cN1 disease at radiotherapy imaging for planning are excluded
- Former radiotherapy, making whole pelvic radiotherapy (WPRT) or PART impossible
- Prior malignancy, not disease-free >5 years, except basocellular skin epithelioma
- Severe or active comorbidity likely to impact the feasibility of WPRT or PART (eg, ulcerative colitis)
- Disorder precluding the understanding of trial information

### Dose Prescription and Treatment Delivery

Dose will be prescribed as D_98_ (ie, the dose received by 98% of the volume and a surrogate for minimal dose) to the PTV of the pelvic LN and PALN. This D_98_ is 45 Gy, to be delivered in 25 fractions of 1.8 Gy. In case of the postprostatectomy situation, the PTV of the prostate and seminal vesicle bed will be treated with a median dose of 70 Gy in 35 fractions. In case of primary radiotherapy to the prostate, the median PTV dose will be 65 Gy in 25 fractions (moderate hypofractionation). Details on dose prescription and constraints for OARs are provided elsewhere [16]. Treatment will be delivered using 6 to 10-MV photons from a linear accelerator (both Elekta and Varian Systems are used). Image-guided radiotherapy is obligatory and will be performed using daily cone-beam CT [35].

### Hormonal Treatment

ADT will be started 2-4 weeks before the start of radiotherapy to overcome the androgen receptor-induced radioresistance [36]. The duration of ADT is 24 months (long term) as all patients belong to a very high-risk population and long-term ADT is the standard of care in these patients [37,38]. Of note, both the use of an luteinizing hormone-releasing hormone-analogue and an antagonist is allowed.

### Primary Endpoint

The primary endpoint is 5-year clinical relapse-free survival (cRFS), defined as the absence of any cR that would be visible at top of the line imaging (see below). Any detected clinical recurrences (r) would be anatomically mapped and categorized as local (rL), pelvic nodal (rN1), retroperitoneal nodal (rM1a), bone (axial, perpendicular, or both, rM1b), or soft-tissue (rM1c). Combinations of different relapse sites are of course possible and will be reported accordingly. Apart from the anatomical site of relapse, the number of relapses, size per relapse, and the subsequent treatment will be recorded.

PSA measurements are performed during follow-up according to a fixed schedule (Table 1). If PSA is undetectable, patients are considered free of cR. In case of biochemical relapse, defined as PSA >0.2 µg/L in the postprostatectomy setting and PSA >nadir+2 µg/L in the primary setting [39,40]. In addition, positron emission tomography-CT (PET-CT) imaging using prostate-specific membrane antigen ligand or ^18^F/^11^C-choline- based PET-CT imaging is acquired. Additional imaging tools include multiparametric MRI and MRI of the axial and perpendicular bones. The decision to perform additional imaging will be taken after multidisciplinary consensus in all cases.

**Table 1:** Standard Protocol Items: Recommendations for Interventional Trials 2013 diagram: Para-Aortic Radiotherapy (PART)-trial.

Timepoint		Study period					
		Enrollment: Screen visit & pre-PART (-t_1_)	Postallocation				
			During PART		Follow-up		
			Weekly	Last day	Month 1, 3, 6, 9, 12	Month 18, 24, 30, 36, 42, 48, 54, 60	Year 6 up to year 10, yearly
**Enrollment**							
	Eligibility screen	X					
	Informed consent	X					
Interventions: Elective PART			—^a^				
**Assessments**							
	Clinical examination	X		X	X	X	X
	Laboratory analysis^b^	X		X	X	X	X
	Registration of pretreatment gastrointestinal and genitourinary morbidities^b^	X					
	Registration quality of life using validated questionnaires	X		X	X	X	X
	Registration of PART-induced toxicity (Common Toxicity Criteria for adverse events)^b^		X	X	X	X	X
	Imaging (after discussion at an interdisciplinary tumor board)	X			X^c^	X^c^	X^c^

^a^Elective PART occurs during the whole period—daily instead of weekly, including the last day.

^b^Standard examinations; laboratory analysis is described in the protocol.

^c^In case of prostate-specific antigen relapse or symptoms.

### Secondary Endpoints

Secondary endpoints are quality of life (QoL), treatment-related acute and late toxicity, time to palliative ADT, time to castration-refractory prostate cancer (CRPC), CSS, and in-field pelvic and para-aortic disease control.

The QoL is measured using the European Organization for Research and Treatment of Cancer (EORTC) core questionnaire [41]. In addition, the EORTC prostate cancer module [42], the EuroQol 5 dimensions questionnaire [43], the International Consultation on Incontinence Short Form score [44], and the International Index of Erectile Function scoring system [45] are used to assess urinary, bowel, and sexual functioning and symptoms and evaluate the side effects of hormonal treatment associated with radiotherapy. QoL questionnaires are handed over to patients before treatment (baseline score) and at well-defined time points (end of treatment; 1 month, 3 months, 6 months, and 9 months after treatment; every 6 months until 5 years after treatment; and every 12 months until 10 years after treatment). QoL results will be presented in accordance with guidelines for reporting health-related QoL outcomes in cancer clinical trials published by the EORTC [46].

Treatment-related toxicity is assessed using the Common Toxicity Criteria for adverse events version 4.0 (CTCAE v4.0) [47]. Abdominal pain, diarrhea, enterocolitis, fecal incontinence, flatulence, hemorrhoids, proctitis, rectal fistula, rectal hemorrhage, rectal pain, noninfectious cystitis, hematuria, urinary frequency, urinary incontinence, urinary retention, urinary tract pain, erectile dysfunction, and fatigue are scored as adverse events according to CTCAE v4.0. Symptoms are scored before treatment. In addition, PART-induced acute toxicity (CTCAE v4.0) is scored weekly during radiation treatment and 1 month and 3 months after treatment. Furthermore, treatment-induced late toxicity (CTCAE v4.0) is scored at 6, 9, and 12 months after treatment; every 6 months until 5 years after treatment; and every 12 months until 10 years after treatment.

Time to palliative ADT is defined as the secondary endpoint of this trial. Indications to initiate palliative ADT are based on the EAU guidelines [39,40] and include the following: PSA>50 µg/L or PSA doubling time <6 months or symptoms due to progressive disease. In case of oligometastatic recurrence (1-3 synchronous metastases), metastasis-directed therapy is the preferential treatment option [48]. Time to CRPC is defined according to the criteria defined in the EAU guidelines [39,40]. CSS is defined as the interval from the date of diagnosis to the date of death from PCa or to the last follow-up date for censoring purposes, if the patient is alive and is still being followed at the time of data cut-off.

### Laboratory Analysis

All laboratory tests are considered standard and include PSA measurement, peripheral blood cell count with formula, kidney function tests, liver function tests, and testosterone measurement. These laboratory tests are done during every follow-up visit.

### Time Schedule

The aim is to recruit the necessary number of patients within a timeframe of 48 months. Follow-up of these patients will be lifelong to correctly estimate the primary and secondary endpoints. Reports on acute PART-induced toxicity and QoL will be expected within 6 months of the closure of the trial. Furthermore, the primary endpoint will be calculated after a median follow-up of 60 months.

### Safety

This project has been funded by “Kom op tegen Kanker” (study number: S59533). The investigators shall report all serious adverse events (grade 3 or higher) immediately to the sponsor. The immediate report shall be followed by detailed, written reports. The immediate and follow-up reports shall identify subjects by code numbers. For reported deaths of a subject, the investigator shall supply the sponsor and the accredited ethics committee with any additional information requested. Patients will be withdrawn from PART if they develop grade 4 toxicity. Based on former experience in cervical cancer, there is negligible chance for occurrence of grade 4 toxicity when PART is delivered [49].

The investigator shall ensure that all relevant information about suspected unexpected serious adverse reactions that are fatal or life threatening is recorded and reported as soon as possible to the minister, and to the competent ethics committee, and in any case, no later than 7 days after knowledge by the investigator of such a case.

All other suspected unexpected serious adverse reactions shall be reported to the minister and the ethics committee concerned as soon as possible but within a maximum of 15 days of first knowledge by the investigator. Furthermore, the principal investigator shall inform other investigators.

### Sample Size and Statistics

We aim to improve 5-year cRFS by 15% (primary endpoint). This would result in a 5-year cRFS of 75% compared with the control group with WPRT only that reaches a 5-year cRFS of 60% [18,20]. For a log-rank test comparing two survival curves with a one-sided significance level of .1, assuming uniform accrual with an accrual time of 48 months and a follow-up time of 12 months, a sample size of 137 is required to obtain a power of at least 80%. Taking into account a dropout of 10%, we aim to include 151 patients. The control group consists of pN1 patients treated with adjuvant ADT and WPRT alone from whom the data have been published before [20]. The incidence of acute and late toxicity will be recorded. In addition, actuarial risk estimates for developing acute and late toxicity will be calculated using Kaplan-Meier analysis. Time to palliative ADT, time to CRPC, CSS, and in-field pelvic and para-aortic disease control will be calculated using Kaplan-Meier actuarial analysis. cRFS, time to CRPC, CSS, and in-field pelvic and para-aortic disease control times are defined from the date of LN dissection until an event or last follow-up. Statistical analysis will be performed using the latest version of SPSS (IBM Corp, Armonk, NY, USA).

## Results

Ethical approval to conduct this study (version 2.0 from December 1, 2016) was granted by the Medical Ethics Committee University Hospitals/Catholic University Leuven (14/12/2016). Written informed consent of patients is mandatory before recruitment. Recruitment of patients started in 2017 and is expected to be completed by March 2020.

As a result of the PART trial, we will publish the 5-year cRFS, defined as the absence of any cR that would be visible at top of the line imaging, our primary endpoint. Furthermore, we will compare these results with the 5-year cRFS of a historical control group of patients who underwent WPRT; the results of this historical control group have already been published by Poelaert et al [20]. This control group includes patients with PCa who underwent PLND and pelvic radiotherapy between January 2000 and January 2016 at a tertiary center (GUH). Those patients were rigorously treated by several authors of this manuscript (GDM, VF, KDC, and NL). A total of 154 pN1 patients with PCa who received WPRT were included. As described in the “Sample size and Statistics” section, survival curves of both groups will be compared using log-rank test. Besides, acute toxicity and QoL results will be published after short-term follow-up, whereas treatment-related late toxicity and QoL results, time to palliative ADT, time to CRPC, CSS, and in-field pelvic and para-aortic disease control will be published after a longer follow-up period.

## Discussion

Currently, management of pN1 PCa is shifting toward a multimodal approach aiming at cure. Several recent studies have shown an improved CSS when adjuvant radiotherapy was added to ADT [13,20,49-51]. Unfortunately, recurrences are still observed. Data suggest relapse at the site of the PALN due to ascending PCa lymphatic spread from the pelvis up to the retroperitoneum in about 75% cases of LN-only recurrence [24,25]. New strategies to further enhance locoregional control while maintaining an acceptable level of toxicity are a possible tool to improve cure rates as locoregional relapse is linked to metastatic progression [52,53]. The use of extended-field IMRT to the PALN plus concurrent cisplatin in cervical cancer improved the outcome for patients with LN-positive stage IB2-IIIB cervical cancer [54]. Based on the evidence that positive LNs are observed before hematogenous spread occurs, we hypothesize that elective para-aortic irradiation will reduce the development of distant metastasis, postpone the need for palliative ADT, and prolong the time to castration-refractory disease.

This protocol describes the design of a nonrandomized phase II trial to evaluate the clinical effectiveness of elective PART using arc therapy for reducing disease recurrence in pN1 patients with PCa. To the best of our knowledge, this is the first phase II trial investigating the benefit of an elective PART in patients with PCa. The results of this study will hopefully provide a sound basis for a prospective randomized phase III study randomizing patients between WPRT only and WPRT with PALN irradiation.

## Appendix

Multimedia Appendix 1 Funding approval (Kom Op Tegen Kanker / Foundation Against Cancer) based on peer review process (Dutch).

Multimedia Appendix 2 Funding approval (Kom Op Tegen Kanker / Foundation against Cancer) based on peer review process (English translation).

## Abbreviations

ADT: androgen deprivation therapy
cR: clinical relapse
cRFS: clinical relapse-free survival
CRPC: castration-refractory prostate cancer
CSS: cause-specific survival
CT: computed tomography
CTCAE: Common Toxicity Criteria for Adverse Events
CTV: clinical target volume
EAU: European Association of Urology
EORTC: European Organization for Research and Treatment of Cancer
ePLND: extended pelvic lymph node dissection
GUH: Ghent University Hospital
IMAT: intensity-modulated arc therapy
LN: lymph node
LUH: Leuven University Hospitals
MRI: magnetic resonance imaging
OARs: organs at risk
PALN: para-aortic lymph node
PART: para-aortic radiotherapy
PCa: prostate cancer
PCSS: prostate cancer-specific survival
PSA: prostate-specific antigen
PTV: planned target volume
QoL: quality of life
RP: radical prostatectomy
VMAT: volume-modulated arc therapy
WPRT: whole pelvic radiotherapy

## References

[1] Siegel RL, Miller KD, Jemal A (2016). Cancer statistics, 2016. CA Cancer J Clin.

[2] Fitzmaurice C, Dicker D, Pain A, Hamavid H, Moradi-Lakeh M, MacIntyre MF, Allen C, Hansen G, Woodbrook R, Wolfe C, Hamadeh RR, Moore A, Werdecker A, Gessner BD, Te AB, McMahon B, Karimkhani C, Yu C, Cooke GS, Schwebel DC, Carpenter DO, Pereira DM, Nash D, Kazi DS, De Leo D, Plass D, Ukwaja KN, Thurston GD, Yun JK, Simard EP, Mills E, Park E, Catal&aacute;-L&oacute;pez Ferr&aacute;n, deVeber G, Gotay C, Khan G, Hosgood HD, Santos IS, Leasher JL, Singh J, Leigh J, Jonas JB, Jonas J, Sanabria J, Beardsley J, Jacobsen KH, Takahashi K, Franklin RC, Ronfani L, Montico M, Naldi L, Tonelli M, Geleijnse J, Petzold M, Shrime MG, Younis M, Yonemoto N, Breitborde N, Yip P, Pourmalek F, Lotufo PA, Esteghamati A, Hankey GJ, Ali R, Lunevicius R, Malekzadeh R, Dellavalle R, Weintraub R, Lucas R, Hay R, Rojas-Rueda D, Westerman R, Sepanlou SG, Nolte S, Patten S, Weichenthal S, Abera SF, Fereshtehnejad S, Shiue I, Driscoll T, Vasankari T, Alsharif U, Rahimi-Movaghar V, Vlassov VV, Marcenes WS, Mekonnen W, Melaku YA, Yano Y, Artaman A, Campos I, MacLachlan J, Mueller U, Kim D, Trillini M, Eshrati B, Williams HC, Shibuya K, Dandona R, Murthy K, Cowie B, Amare AT, Antonio CA, Casta&ntilde;eda-Orjuela C, van Gool CH, Violante F, Oh I, Deribe K, Soreide K, Knibbs L, Kereselidze M, Green M, Cardenas R, Roy N, Tillmann T, Tillman T, Li Y, Krueger H, Monasta L, Dey S, Sheikhbahaei S, Hafezi-Nejad N, Kumar GA, Sreeramareddy CT, Dandona L, Wang H, Vollset SE, Mokdad A, Salomon JA, Lozano R, Vos T, Forouzanfar M, Lopez A, Murray C, Naghavi M, Global Burden of Disease Cancer Collaboration (2015). The Global Burden of Cancer 2013. JAMA Oncol.

[3] Roach M, Marquez C, Yuo HS, Narayan P, Coleman L, Nseyo UO, Navvab Z, Carroll PR (1994). Predicting the risk of lymph node involvement using the pre-treatment prostate specific antigen and Gleason score in men with clinically localized prostate cancer. Int J Radiat Oncol Biol Phys.

[4] Briganti A, Larcher A, Abdollah F, Capitanio U, Gallina A, Suardi N, Bianchi M, Sun M, Freschi M, Salonia A, Karakiewicz PI, Rigatti P, Montorsi F (2012). Updated nomogram predicting lymph node invasion in patients with prostate cancer undergoing extended pelvic lymph node dissection: the essential importance of percentage of positive cores. Eur Urol.

[5] Hansen J, Rink M, Bianchi M, Kluth LA, Tian Z, Ahyai SA, Shariat SF, Briganti A, Steuber T, Fisch M, Graefen M, Karakiewicz PI, Chun FK (2013). External validation of the updated Briganti nomogram to predict lymph node invasion in prostate cancer patients undergoing extended lymph node dissection. Prostate.

[6] Mottet N, Bellmunt J, Bolla M, Briers E, Cumberbatch MG, De Santis M, Fossati N, Gross T, Henry AM, Joniau S, Lam TB, Mason MD, Matveev VB, Moldovan PC, van den Bergh RCN, Van den Broeck T, van der Poel HG, van der Kwast TH, Rouvi&egrave;re Olivier, Schoots IG, Wiegel T, Cornford P (2017). EAU-ESTRO-SIOG Guidelines on Prostate Cancer. Part 1: Screening, Diagnosis, and Local Treatment with Curative Intent. Eur Urol.

[7] Briganti A, Blute ML, Eastham JH, Graefen M, Heidenreich A, Karnes JR, Montorsi F, Studer UE (2009). Pelvic lymph node dissection in prostate cancer. Eur Urol.

[8] Schumacher MC, Burkhard FC, Thalmann GN, Fleischmann A, Studer UE (2008). Good outcome for patients with few lymph node metastases after radical retropubic prostatectomy. Eur Urol.

[9] Schiavina R, Bertaccini A, Franceschelli A, Manferrari F, Vagnoni V, Borghesi M, Morselli-Labate AM, Martorana G (2010). The impact of the extent of lymph-node dissection on biochemical relapse after radical prostatectomy in node-negative patients. Anticancer Res.

[10] Messing EM, Manola J, Yao J, Kiernan M, Crawford D, Wilding G, di'SantAgnese PA, Trump D, Eastern Cooperative Oncology Group study EST 3886 (2006). Immediate versus deferred androgen deprivation treatment in patients with node-positive prostate cancer after radical prostatectomy and pelvic lymphadenectomy. Lancet Oncol.

[11] Gakis G, Boorjian SA, Briganti A, Joniau S, Karazanashvili G, Karnes RJ, Mattei A, Shariat SF, Stenzl A, Wirth M, Stief CG (2014). The role of radical prostatectomy and lymph node dissection in lymph node-positive prostate cancer: a systematic review of the literature. Eur Urol.

[12] Abdollah F, Gandaglia G, Suardi N, Capitanio U, Salonia A, Nini A, Moschini M, Sun M, Karakiewicz PI, Shariat SF, Montorsi F, Briganti A (2015). More extensive pelvic lymph node dissection improves survival in patients with node-positive prostate cancer. Eur Urol.

[13] Abdollah F, Karnes RJ, Suardi N, Cozzarini C, Gandaglia G, Fossati N, Vizziello D, Sun M, Karakiewicz PI, Menon M, Montorsi F, Briganti A (2014). Impact of adjuvant radiotherapy on survival of patients with node-positive prostate cancer. J Clin Oncol.

[14] Wong AT, Schwartz D, Osborn V, Safdieh J, Weiner J, Schreiber D (2016). Adjuvant radiation with hormonal therapy is associated with improved survival in men with pathologically involved lymph nodes after radical surgery for prostate cancer. Urol Oncol.

[15] Tosco L, Laenen A, Briganti A, Gontero P, Karnes RJ, Bastian PJ, Chlosta P, Claessens F, Chun FK, Everaerts W, Gratzke C, Albersen M, Graefen M, Kneitz B, Marchioro G, Salas RS, Tombal B, Van den Broeck T, Van Der Poel H, Walz J, De Meerleer G, Bossi A, Haustermans K, Van Poppel H, Spahn M, Joniau S, European Multicenter Prostate Cancer ClinicalTranslational Research group (EMPaCT) (2017). The EMPaCT Classifier: A Validated Tool to Predict Postoperative Prostate Cancer-related Death Using Competing-risk Analysis. Eur Urol Focus.

[16] Fonteyne V, De Gersem W, De Neve W, Jacobs F, Lumen N, Vandecasteele K, Villeirs G, De Meerleer G (2009). Hypofractionated intensity-modulated arc therapy for lymph node metastasized prostate cancer. Int J Radiat Oncol Biol Phys.

[17] Infusino E (2015). Clinical utility of RapidArc™ radiotherapy technology. Cancer Manag Res.

[18] Van Hemelryk A, De Meerleer G, Ost P, Poelaert F, De Gersem W, Decaestecker K, De Visschere P, Fonteyne V (2016). The Outcome for Patients With Pathologic Node-Positive Prostate Cancer Treated With Intensity Modulated Radiation Therapy and Androgen Deprivation Therapy: A Case-Matched Analysis of pN1 and pN0 Patients. Int J Radiat Oncol Biol Phys.

[19] Van Praet C, Ost P, Lumen N, De Meerleer G, Vandecasteele K, Villeirs G, Decaestecker K, Fonteyne V (2013). Postoperative high-dose pelvic radiotherapy for N+ prostate cancer: toxicity and matched case comparison with postoperative prostate bed-only radiotherapy. Radiother Oncol.

[20] Poelaert F, Fonteyne V, Ost P, De Troyer B, Decaestecker K, De Meerleer G, De Visschere P, Claeys T, Dhondt B, Lumen N (2017). Whole pelvis radiotherapy for pathological node-positive prostate cancer : Oncological outcome and prognostic factors. Strahlenther Onkol.

[21] Daneshmand S, Quek ML, Stein JP, Lieskovsky G, Cai J, Pinski J, Skinner EC, Skinner DG (2004). Prognosis of patients with lymph node positive prostate cancer following radical prostatectomy: long-term results. J Urol.

[22] Boorjian SA, Thompson RH, Siddiqui S, Bagniewski S, Bergstralh EJ, Karnes RJ, Frank I, Blute ML (2007). Long-term outcome after radical prostatectomy for patients with lymph node positive prostate cancer in the prostate specific antigen era. J Urol.

[23] Griebling TL, Ozkutlu D, See WA, Cohen MB (1997). Prognostic implications of extracapsular extension of lymph node metastases in prostate cancer. Mod Pathol.

[24] Rigatti P, Suardi N, Briganti A, Da Pozzo LF, Tutolo M, Villa L, Gallina A, Capitanio U, Abdollah F, Scattoni V, Colombo R, Freschi M, Picchio M, Messa C, Guazzoni G, Montorsi F (2011). Pelvic/retroperitoneal salvage lymph node dissection for patients treated with radical prostatectomy with biochemical recurrence and nodal recurrence detected by [11C]choline positron emission tomography/computed tomography. Eur Urol.

[25] Briganti A, Suardi N, Capogrosso P, Passoni N, Freschi M, di Trapani E, Gallina A, Capitanio U, Abdollah F, Tutolo M, Bianchi M, Salonia A, Da Pozzo LF, Montorsi F, Rigatti P (2012). Lymphatic spread of nodal metastases in high-risk prostate cancer: The ascending pathway from the pelvis to the retroperitoneum. Prostate.

[26] Rischke HC, Schultze-Seemann W, Wieser G, Krönig M, Drendel V, Stegmaier P, Krauss T, Henne K, Volegova-Neher N, Schlager D, Kirste S, Grosu A, Jilg CA (2015). Adjuvant radiotherapy after salvage lymph node dissection because of nodal relapse of prostate cancer versus salvage lymph node dissection only. Strahlenther Onkol.

[27] Brierley J, Gospodarowicz M, Wittekind C (2017). TNM classification of malignant tumours 8th Edition. Oxford, John Wiley & sons.

[28] Joniau S, Tosco L, Briganti A, Vanden BT, Gontero P, Karnes RJ, Spahn M, Van PH, European Multicenter Prostate Cancer ClinicalTranslational research group (2013). Results of surgery for high-risk prostate cancer. Curr Opin Urol.

[29] Harris VA, Staffurth J, Naismith O, Esmail A, Gulliford S, Khoo V, Lewis R, Littler J, McNair H, Sadoyze A, Scrase C, Sohaib A, Syndikus I, Zarkar A, Hall E, Dearnaley D, PIVOTAL Trialists (2015). Consensus Guidelines and Contouring Atlas for Pelvic Node Delineation in Prostate and Pelvic Node Intensity Modulated Radiation Therapy. Int J Radiat Oncol Biol Phys.

[30] Poortmans P, Bossi A, Vandeputte K, Bosset M, Miralbell R, Maingon P, Boehmer D, Budiharto T, Symon Z, van den Bergh AC, Scrase C, Van Poppel H, Bolla M, EORTC Radiation Oncology Group (2007). Guidelines for target volume definition in post-operative radiotherapy for prostate cancer, on behalf of the EORTC Radiation Oncology Group. Radiother Oncol.

[31] Ost P, De Meerleer G, Vercauteren T, De Gersem W, Veldeman L, Vandecasteele K, Fonteyne V, Villeirs G (2011). Delineation of the postprostatectomy prostate bed using computed tomography: interobserver variability following the EORTC delineation guidelines. Int J Radiat Oncol Biol Phys.

[32] Villeirs GM, Van Vaerenbergh K, Vakaet L, Bral S, Claus F, De Neve WJ, Verstraete KL, De Meerleer GO (2005). Interobserver delineation variation using CT versus combined CT + MRI in intensity-modulated radiotherapy for prostate cancer. Strahlenther Onkol.

[33] De Meerleer GO, Villeirs GM, Vakaet L, Delrue LJ, De Neve WJ (2004). The incidence of inclusion of the sigmoid colon and small bowel in the planning target volume in radiotherapy for prostate cancer. Strahlenther Onkol.

[34] Vandecasteele K, De Neve W, De Gersem W, Delrue L, Paelinck L, Makar A, Fonteyne V, De Wagter C, Villeirs G, De Meerleer G (2009). Intensity-modulated arc therapy with simultaneous integrated boost in the treatment of primary irresectable cervical cancer. Treatment planning, quality control, and clinical implementation. Strahlenther Onkol.

[35] Ost P, De Meerleer G, De Gersem W, Impens A, De Neve W (2011). Analysis of prostate bed motion using daily cone-beam computed tomography during postprostatectomy radiotherapy. Int J Radiat Oncol Biol Phys.

[36] Spratt DE, Evans MJ, Davis BJ, Doran MG, Lee MX, Shah N, Wongvipat J, Carnazza KE, Klee GG, Polkinghorn W, Tindall DJ, Lewis JS, Sawyers CL (2015). Androgen Receptor Upregulation Mediates Radioresistance after Ionizing Radiation. Cancer Res.

[37] Bolla M, de Reijke TM, Van Tienhoven G, Van den Bergh AC, Oddens J, Poortmans PMP, Gez E, Kil P, Akdas A, Soete G, Kariakine O, van der Steen-Banasik EM, Musat E, Pi&eacute;rart M, Mauer ME, Collette L, EORTC Radiation Oncology GroupGenito-Urinary Tract Cancer Group (2009). Duration of androgen suppression in the treatment of prostate cancer. N Engl J Med.

[38] Horwitz EM, Bae K, Hanks GE, Porter A, Grignon DJ, Brereton HD, Venkatesan V, Lawton CA, Rosenthal SA, Sandler HM, Shipley WU (2008). Ten-year follow-up of radiation therapy oncology group protocol 92-02: a phase III trial of the duration of elective androgen deprivation in locally advanced prostate cancer. J Clin Oncol.

[39] Heidenreich A, Bastian PJ, Bellmunt J, Bolla M, Joniau S, van der Kwast T, Mason M, Matveev V, Wiegel T, Zattoni F, Mottet N, European Association of Urology (2014). EAU guidelines on prostate cancer. Part II: Treatment of advanced, relapsing, and castration-resistant prostate cancer. Eur Urol.

[40] Cornford P, Bellmunt J, Bolla M, Briers E, De Santis M, Gross T, Henry AM, Joniau S, Lam TB, Mason MD, van der Poel HG, van der Kwast TH, Rouvi&egrave;re Olivier, Wiegel T, Mottet N (2017). EAU-ESTRO-SIOG Guidelines on Prostate Cancer. Part II: Treatment of Relapsing, Metastatic, and Castration-Resistant Prostate Cancer. Eur Urol.

[41] Aaronson NK, Ahmedzai S, Bergman B, Bullinger M, Cull A, Duez NJ, Filiberti A, Flechtner H, Fleishman SB, de Haes JC (1993). The European Organization for Research and Treatment of Cancer QLQ-C30: a quality-of-life instrument for use in international clinical trials in oncology. J Natl Cancer Inst.

[42] Borghede G, Sullivan M (1996). Measurement of quality of life in localized prostatic cancer patients treated with radiotherapy. Development of a prostate cancer-specific module supplementing the EORTC QLQ-C30. Qual Life Res.

[43] Herdman M, Gudex C, Lloyd A, Janssen M, Kind P, Parkin D, Bonsel G, Badia X (2011). Development and preliminary testing of the new five-level version of EQ-5D (EQ-5D-5L). Qual Life Res.

[44] Avery K, Donovan J, Peters TJ, Shaw C, Gotoh M, Abrams P (2004). ICIQ: a brief and robust measure for evaluating the symptoms and impact of urinary incontinence. Neurourol Urodyn.

[45] Rosen RC, Cappelleri JC, Smith MD, Lipsky J, Peña BM (1999). Development and evaluation of an abridged, 5-item version of the International Index of Erectile Function (IIEF-5) as a diagnostic tool for erectile dysfunction. Int J Impot Res.

[46] Bottomley A, Flechtner H, Efficace F, Vanvoorden V, Coens C, Therasse P, Velikova G, Blazeby J, Greimel E, European Organisation for ResearchTreatment of Cancer (EORTC) Data CenterQuality of Life Group (2005). Health related quality of life outcomes in cancer clinical trials. Eur J Cancer.

[47] (2009). European Organisation for Research and Treatment of Cancer.

[48] Decaestecker K, De Meerleer G, Lambert B, Delrue L, Fonteyne V, Claeys T, De Vos F, Huysse W, Hautekiet A, Maes G, Ost P (2014). Repeated stereotactic body radiotherapy for oligometastatic prostate cancer recurrence. Radiat Oncol.

[49] Da Pozzo LF, Cozzarini C, Briganti A, Suardi N, Salonia A, Bertini R, Gallina A, Bianchi M, Fantini GV, Bolognesi A, Fazio F, Montorsi F, Rigatti P (2009). Long-term follow-up of patients with prostate cancer and nodal metastases treated by pelvic lymphadenectomy and radical prostatectomy: the positive impact of adjuvant radiotherapy. Eur Urol.

[50] Briganti A, Karnes RJ, Da Pozzo LF, Cozzarini C, Capitanio U, Gallina A, Suardi N, Bianchi M, Tutolo M, Salonia A, Di MN, Rigatti P, Montorsi F, Blute M (2011). Combination of adjuvant hormonal and radiation therapy significantly prolongs survival of patients with pT2-4 pN+ prostate cancer: results of a matched analysis. Eur Urol.

[51] James ND, Spears MR, Clarke NW, Dearnaley DP, Mason MD, Parker CC, Ritchie AWS, Russell JM, Schiavone F, Attard G, de Bono JS, Birtle A, Engeler DS, Elliott T, Matheson D, O'Sullivan J, Pudney D, Srihari N, Wallace J, Barber J, Syndikus I, Parmar MKB, Sydes MR, STAMPEDE Investigators (2016). Failure-Free Survival and Radiotherapy in Patients With Newly Diagnosed Nonmetastatic Prostate Cancer: Data From Patients in the Control Arm of the STAMPEDE Trial. JAMA Oncol.

[52] Coen JJ, Zietman AL, Thakral H, Shipley WU (2002). Radical radiation for localized prostate cancer: local persistence of disease results in a late wave of metastases. J Clin Oncol.

[53] Zelefsky MJ, Yamada Y, Fuks Z, Zhang Z, Hunt M, Cahlon O, Park J, Shippy A (2008). Long-term results of conformal radiotherapy for prostate cancer: impact of dose escalation on biochemical tumor control and distant metastases-free survival outcomes. Int J Radiat Oncol Biol Phys.

[54] Liang J, Chen S, Hung Y, Yeh L, Chang W, Lin W, Chang Y (2014). Low-dose, prophylactic, extended-field, intensity-modulated radiotherapy plus concurrent weekly cisplatin for patients with stage IB2-IIIB cervical cancer, positive pelvic lymph nodes, and negative para-aortic lymph nodes. Int J Gynecol Cancer.

