# Supplementary material for: Benefits of Elective Para-Aortic Radiotherapy for pN1 Prostate Cancer Using Arc Therapy (Intensity-Modulated or Volumetric Modulated Arc Therapy): Protocol for a Nonrandomized Phase II Trial
Source: JMIR Res Protoc. 2018 Dec 13;7(12):e11256. doi: 10.2196/11256 (PMC6315267; doi:10.2196/11256)
Supplement: Multimedia Appendix 1 [file resprot_v7i12e11256_app1.pdf]

Kom op tegen Kanker

OVEREENKOMST FINANCIERING ONDERZOEKSPROJECT
KOM OP TEGEN KANKER

TUSSEN DE ONDERGETEKENDEN, PARTIJEN BIJ DEZE OVEREENKOMST:

KOM OP TEGEN KANKER-VZW

met zetel te Koningsstraat 217

1210 - Brussel

K.B.O. nr. 0442.528.054

hierbij rechtsgeldig vertegenwoordigd door

Marc Michils,

Directeur

hierna genoemd de "projectfinancier"

en

de instelling Universitaire Ziekenhuizen Leuven, K.B.O. nr. 419.052.173, hierna de "instelling", vertegenwoordigd door prof. dr. Marc Decramer, die prof. dr. Gert De Meerleer aanduidt als verantwoordelijke binnen de instelling voor de uitvoering van dit contract, hierna genoemd "de projectverantwoordelijke". De instelling en de projectverantwoordelijke samen worden 'medecontractanten' genoemd.

WORDT VOORAFGAAND UITEENGEZET WAT VOLGT:

De projectfinancier stelt gelden beschikbaar voor de financiering van wetenschappelijk onderzoek dat bijdraagt tot een verbetering van de overlevingskansen en de levenskwaliteit van kankerpatiënten.

De projectverantwoordelijke heeft een project ingediend dat na beoordeling werd goedgekeurd.

De aan het project toegekende toelage wordt gefinancierd met de opbrengst uit legaten en erfenissen evenals de acties van de projectfinancier. Ze is maar mogelijk dankzij het vertrouwen dat de erflaters stellen in de strijd die de projectfinancier tegen kanker voert. In dit kader moeten de aanvragers er bijgevolg nauwgezet op toezien dat de toegekende middelen zo efficiënt mogelijk worden besteed met het

oog op de realisatie van het doel dat de erflaters en donateurs voor ogen hadden, namelijk: de verbetering van de overlevingskansen en de levenskwaliteit van kankerpatiënten.

WORDT OVEREENGEKOMEN WAT VOLGT:

Artikel 1. Voorwerp

1.1. Binnen de bij deze overeenkomst vastgelegde modaliteiten verklaart de projectfinancier zich akkoord om een projecttoelage aan de instelling toe te kennen ter realisatie van het door de projectverantwoordelijke ingediende en goedgekeurde project, zoals omschreven in bijlage 1 en verder in deze overeenkomst aangeduid als "*het project*".

1.2. De projectverantwoordelijke verbindt er zich toe het project uit te voeren conform de in bijlage 1 goedgekeurde projectdocumenten, met name de projectaanvraag, eventueel de bijkomende documenten die tijdens de beoordelingsprocedure werden opgevraagd bij de projectverantwoordelijke, het financieel plan en de bijzondere modaliteiten bepaald in deze overeenkomst.

1.3. Deze overeenkomst doet op geen enkele wijze enige arbeidsrechtelijke band ontstaan tussen de projectfinancier en de projectverantwoordelijke.

Artikel 2. Projectbeheer

2.1. Het projectbeheer wordt voor de instelling waargenomen door de projectverantwoordelijke en de vertegenwoordiger van de instelling. De projectverantwoordelijke en de vertegenwoordiger van de instelling zijn twee verschillende personen binnen de instelling.

- De projectverantwoordelijke is verantwoordelijk voor de goede uitvoering van het project, staat ervoor in dat de vastgelegde resultaatsgebieden gehaald worden, staat in voor het correcte beheer van de toegewezen projectgelden, staat ervoor in dat de gelden uitsluitend aangewend worden voor de budgetposten zoals vastgelegd in het projectvoorstel, en kan te allen tijde een gedetailleerd overzicht van de gemaakte uitgaven aan de projectfinancier geven.
- De vertegenwoordiger van de instelling is bevoegd om de instelling rechtsgeldig te verbinden bij de uitvoering van het project dat het voorwerp uitmaakt van deze overeenkomst. Hij ziet toe op het correcte beheer van de toegewezen projectgelden, waakt erover dat de gelden uitsluitend aangewend worden voor de doeleinden zoals vermeld in het projectvoorstel. Hij duidt de projectverantwoordelijke aan als de persoon die binnen de instelling verantwoordelijk is voor de uitvoering van het project.

De projectverantwoordelijke en de verantwoordelijke van de instelling worden bepaald in bijlage 1 bij deze overeenkomst.

Elke wijziging van projectverantwoordelijke en verantwoordelijke van de instelling wordt voorafgaand schriftelijk aan de projectfinancier meegedeeld.

2.2. De overeenkomst wordt zowel door de projectverantwoordelijke, als door de instelling ondertekend.

2.3. De medecontractanten zijn ten opzichte van de projectfinancier hoofdelijk aansprakelijk voor alle verbintenissen uit deze overeenkomst.

Artikel 3. Aanvang en duur project

3.1. Het project start op 15/11/2016. Dit contract wordt gesloten onder de opschortende voorwaarde dat het onderzoek wordt goedgekeurd door de ethische commissie.

3.2. De termijn van het project zal 48 maanden bedragen, waardoor het ten laatste een einde zal nemen op 14/11/2020.

3.3. Van zowel de startdatum, de einddatum als de termijn kan niet eenzijdig door de projectverantwoordelijke worden afgeweken, behoudens voorafgaand schriftelijk akkoord van de projectfinancier. Elke overschrijding van de vooropgestelde termijn dient in elk geval op een gemotiveerde wijze onverwijld schriftelijk aan de projectfinancier te worden aangevraagd.

Artikel 4. Projecttoelage

4.1. De projectfinancier stelt ter realisatie van het project een projecttoelage ten bedrage van maximaal 269.304 euro ter beschikking aan de instelling.

De projecttoelage betreft een subsidie voor een project dat door de projectverantwoordelijke bij de projectfinancier werd ingediend. De projectfinancier is voor de subsidiëring van dit project geen BTW verschuldigd.

4.2. De projectverantwoordelijke bevestigt dat de voorziene steun in de overeenkomst voldoende is om het project uit te voeren en er voldoende personeel kan vrijgesteld worden om het project uit te voeren.

4.3. De projecttoelage kan enkel aangewend worden overeenkomstig de budgetposten nader omschreven in het in bijlage 1 opgenomen financieel plan ter verwezenlijking van de activiteiten die in de projectaanvraag worden bepaald en voor de termijn die in deze overeenkomst is afgesproken.

Voor elke afwijking van de budgetposten nader bepaald in het oorspronkelijk ingediende financieel plan, moet de projectverantwoordelijke bij de projectfinancier schriftelijk een aanvraag tot herziening indienen. Enkel wanneer de projectfinancier een aangevraagde herziening schriftelijk goedkeurt, kan de wijziging en bijbehorende betaling worden uitgevoerd.

De projectverantwoordelijke waarborgt dat er met de toegekende toelage in geen geval overheadkosten of algemene werkingskosten van de instelling (b.v. huur gebouw, elektriciteit, water, verwarming,...) die het project uitvoert, worden betaald.

De projecttoelage kan niet worden aangewend voor de vergoeding van BTW. Uitzondering hierop kan gemaakt worden door de projectfinancier indien aan volgende 2 voorwaarden tezamen voldaan is:

a. Het gaat om BTW die niet terugvorderbaar of niet verrekenbaar is met de BTW Administratie

EN

b. Deze btw niet had kunnen vermeden worden omdat er binnen de doelstelling van de organisatie van de projectverantwoordelijke geen alternatieven zijn/waren om de onderliggende kosten zonder BTW ten laste van het project te leggen.

Voorbeeld 1: Het in dienst nemen van een tijdelijke medewerker langs een interim-bureau en aldus niet recupereerbare BTW genereert, had kunnen vermeden worden door het rechtstreeks in dienst nemen van de medewerker middels een tijdelijke arbeidsovereenkomst.

Voorbeeld 2: Een onderzoeksproject wordt toegekend aan organisatie A maar wordt maar deels door organisatie A zelf uitgevoerd en deels door organisatie B. Indien het transfereren van financieringsfondsen van A naar B (voor de remuneratie van B) gepaard gaat met het genereren van niet recupereerbare BTW in de schoot van partij B, dan had dit vermeden kunnen worden door het opmaken van de financieringsovereenkomst met 2 begunstigden (A en B) in plaats van A alleen.

4.4. Indien het project voortijdig wordt stopgezet of niet integraal kan worden gerealiseerd of indien de toelage niet integraal moet worden besteed om de geplande activiteiten te realiseren, dan dient de projectverantwoordelijke de projectfinancier hier steeds onmiddellijk schriftelijk van op de hoogte te brengen. Het reeds betaalde maar niet benutte deel van de projecttoelage wordt in zulk geval door de instelling onverwijld teruggestort. Overeenkomstig de bepalingen in artikel 9 kan ook het benutte deel van de reeds betaalde toelage door de projectfinancier worden teruggevorderd.

4.5. De projecttoelage wordt in principe overeenkomstig de in artikel 5 van deze overeenkomst bepaalde regels in delen uitbetaald, waarbij de eerste delen in de regel in maandelijkse schijven worden uitgekeerd. Het laatste deel, zijnde de laatste schijf van 10% van de projecttoelage, wordt in één keer uitgekeerd na goedkeuring van het eindverslag door de projectfinancier.

4.6. De instelling zal op het eerste verzoek alle nodige uitleg, facturen en andere bewijsstukken verschaffen die verband houden met de aanwending van de projecttoelage.

De projectfinancier behoudt zich eveneens het recht om op gelijk welk moment in de looptijd van het project een expert aan te stellen voor nazicht van de financiële stand van zaken van het project.

4.7. Indien er in de loop van het project bijkomende financiering van andere instanties verworven wordt of indien de projectverantwoordelijke of de instelling zelf geldinzamelingsacties start voor bijkomende financiering van het project, dan dient de projectfinancier hier meteen schriftelijk van op de hoogte gebracht te worden.

4.8. Indien de projectverantwoordelijke of de instelling zelf in de loop van het project bijkomende steun van de overheid of een andere instantie krijgt voor de realisatie van hetzelfde project, dan zal het deel van de toegekende toelage dat overlapt met de bijkomende overheidssteun worden teruggevraagd.

4.9. Indien in de loop van het project zich nieuwe mogelijkheden voordoen om steun te verwerven bij de overheid voor de realisatie van hetzelfde project, dan is de projectverantwoordelijke of de instelling zelf, ertoe gehouden om alles in het werk te stellen om deze steun te verwerven. Indien deze steun met succes wordt verworven dan zal het deel van de toegekende toelage dat overlapt met de bijkomende steun worden teruggevraagd.

Artikel 5. Evaluatie- en betalingsmodaliteiten

5.1. Tijdens de duur van het project zijn vooraf bepaalde evaluatiemomenten ingebouwd die de projectverantwoordelijke nauwgezet dient na te leven.

De verdere toekenning van de in artikel 4 bepaalde projecttoelage wordt afhankelijk gesteld van deze evaluatiemomenten.

5.2. Voor projecten die langer dan een jaar lopen of die maximum één jaar lopen maar een toelage van minstens 25.000 euro vereisen, dient de projectverantwoordelijke om aanspraak te maken op de volgende schijf van de toegekende projecttoelage, jaarlijks een aanvraag tot voortzetting in te dienen bij de projectfinancier.

Deze jaarlijkse aanvraag tot voortzetting omvat steeds een verslag van de vooruitgang van het project op inhoudelijk vlak en op financieel vlak, evenals een planning voor de komende periode met de nodige toelichting. Het verslag geeft een duidelijk overzicht van wat was vooropgesteld en wat ondertussen gerealiseerd werd per resultaatsgebied en per budgetpost. Bijzondere aandacht wordt hierbij besteed aan de eventuele knelpunten.

Pas na evaluatie en goedkeuring door de projectfinancier van dit verslag zal de uitbetaling van de volgende schijf van de projecttoelage starten.

5.3. Voor projecten die maximum een jaar lopen en een toelage ontvangen lager dan 25.000 euro, bestaat de aanvraag in de helft van het project in afwijking van het bepaalde in artikel 5.2. van deze overeenkomst, uit een beknopt verslag waaruit de goede voortgang van het project en de eventuele knelpunten blijken.

5.4. Ten laatste tegen 14/11/2017, 14/11/2018 en 14/11/2019 bezorgt de projectverantwoordelijke een aanvraag om het project verder te zetten.

Enkel in zeer uitzonderlijke omstandigheden en mits voorafgaande schriftelijke toestemming kan van deze termijn worden afgeweken.

5.5. Los van de in dit artikel bepaalde rapportering verbindt de projectverantwoordelijke zich ertoe om knelpunten die zich in de loop van het project zouden (kunnen) voordoen onmiddellijk schriftelijk aan de projectfinancier te melden.

5.6. De projectfinancier kan bovendien te allen tijde aan de projectverantwoordelijke en de instelling om een schriftelijk verslag vragen aangaande de planning, de vooruitgang en de resultaten van het project en/of aangaande de in het kader van het project gemaakte uitgaven.

5.7. De projectfinancier behoudt zich eveneens het recht voor om op elk moment in de looptijd van het project een overleg te organiseren met oog op tussentijdse evaluatie betreffende de vooruitgang van het project. Als blijkt dat het project niet voldoende vooruitgang maakt, wordt door de projectfinancier een vergadering belegd voor bijsturing met de projectverantwoordelijke en met de instelling. Voortzetting van het project is hierna enkel mogelijk als beide partijen het eens zijn over de verdere aanpak. Afspraken i.v.m. de eventuele bijsturing van het project worden op dat moment als bijlage bij deze overeenkomst gevoegd.

5.8. Ten laatste drie maanden na het einde van de projecttermijn, zijnde uiterlijk op 14/02/2021, wordt een eindverslag ingediend. Overeenkomstig artikel 4.5. wordt na goedkeuring van het eindverslag door de projectfinancier de laatste schijf van 10% van de projecttoelage uitbetaald. Enkel in zeer uitzonderlijke omstandigheden en mits voorafgaande schriftelijke toestemming kan van deze termijn worden afgeweken.

Dit verslag geeft een overzicht van hoe de vooropgestelde doelstellingen bereikt zijn, met specifieke informatie over bereikte resultaten (per resultaatsgebied), de bereikte doelgroep(en) en hoe het project aan hun noden tegemoet kwam, en bijgevoegd de output van het project (brochures, rapporten, werkinstrumenten, lijst met gerealiseerde publicaties,...). Het tweede deel van dit eindverslag bevat een gedetailleerd financieel overzicht van het project met in bijlage de financiële bewijsstukken voor de gemaakte kosten. De projectfinancier zal nagaan of de toelage effectief werd besteed aan de activiteiten zoals in het projectvoorstel gespecificeerd. Daartoe kan de projectfinancier bijkomende informatie opvragen wanneer er onduidelijkheden zijn in het eindverslag.

Artikel 6. Resultaat van het onderzoek – intellectuele eigendom

De projectverantwoordelijke heeft het recht om in de loop van het project materiaal van het project vallende onder deze overeenkomst te publiceren. Hij heeft het recht om na de beëindiging van het project naar eigen goeddunken verder het materiaal resulterend uit het project te publiceren in wetenschappelijke tijdschriften en op wetenschappelijke conferenties.

De projectverantwoordelijke bezorgt de projectfinancier na afloop van het project een lijst met de publicaties die in het kader van het project werden gerealiseerd evenals een elektronisch exemplaar van deze publicaties. Hij houdt de projectfinancier op de hoogte van de publicaties die in het kader van het project nog de daarop volgende jaren worden gerealiseerd.

Mits uitdrukkelijke voorafgaande toestemming van de projectverantwoordelijke kan de projectfinancier de resultaten en de teksten van het onderzoek geheel of gedeeltelijk publiceren, via om het even welk medium, of meedelen aan derden, zonder enige vergoeding.

De eventuele commercialisering en de intellectuele eigendomsrechten blijven uitsluitend in handen van de projectverantwoordelijke en de instelling, tenzij andersluidende overeenkomst met de projectfinancier.

Artikel 7. Communicatie

De projectverantwoordelijke verbindt er zich toe om in alle publieke mededelingen en publicaties betreffende het project de steun van de projectfinancier te vermelden. Tenzij anders voorafgaand en schriftelijk werd overeengekomen, zal enkel volgende formule worden gebruikt: "onderzoeksproject gerealiseerd met de steun van Kom op tegen Kanker".

De projectverantwoordelijke verklaart zich bereid om zijn medewerking te verlenen aan initiatieven die door de projectfinancier worden opgezet om de onderzoeksfinanciering van de projectfinancier beter bekend te maken bij het grote publiek of bij potentiële donateurs, en aan initiatieven om bestaande donateurs te informeren over het lopende onderzoeksproject.

De projectverantwoordelijke verklaart zich bereid om de resultaten van het onderzoeksproject bekend te maken op een gezamenlijke persconferentie met Kom op tegen Kanker.

Artikel 8. Verantwoordelijkheid van de projectverantwoordelijke

De projectverantwoordelijke is verantwoordelijk voor het naleven van de afspraken en deadlines die in deze overeenkomst worden overeengekomen, voor het tijdig indienen van de vereiste documenten (aanvraag tot voortzetting van het project, eindverslag,...) in de loop van het project, alsook om eventueel een herziening van het oorspronkelijke inhoudelijke of financiële plan aan te vragen.

Aanvragen tot voortzetting of herziening evenals eindverslagen worden geüpload in het projectbeheersysteem van Kom op tegen Kanker toegankelijk via de website www.komoptegenkanker.be. In elk zal ontvangst slechts gelden indien het toegankelijk is voor de projectbeheerder via het projectbeheersysteem.

Artikel 9. Niet-naleving verbintenissen

Onverminderd de mogelijkheid om deze overeenkomst overeenkomstig artikel 10 eenzijdig te ontbinden en onverminderd de mogelijkheid tot het bekomen van schadevergoeding, kan de projectfinancier de betaling van de projecttoelage zonder voorafgaande ingebrekestelling opschorten, de projecttoelage wijzigen en/of de reeds toegekende projecttoelage (al dan niet gedeeltelijk) terugvorderen bij niet-naleving van de in deze overeenkomst of in de bijlage bepaalde verbintenissen, zoals:

- het niet of het inhoudelijk niet conform uitvoeren van het project;
- het niet naleven van de overeengekomen timing;
- het niet of onvoldoende verstrekken van informatie en/of inzage in de relevante projectgegevens;
- het niet correct besteden van de projecttoelage zoals in het oorspronkelijk ingediende financieel plan is voorzien.
- het niet vermelden van de afgesproken vermelding uit artikel 6 bij publiciteit;

Bij een dergelijke beslissing zullen de projectverantwoordelijke en de instelling hiervan schriftelijk en met redenen omkleed op de hoogte worden gebracht.

Artikel 10. Ontbinding van de overeenkomst

Onverminderd de mogelijkheid tot het bekomen van schadevergoeding, heeft de projectfinancier het recht om deze overeenkomst zonder voorafgaande ingebrekestelling met onmiddellijke ingang en zonder enige schadeloosstelling voor de projectverantwoordelijke of de instelling eenzijdig te beëindigen bij volgende ernstige contractuele tekortkomingen:

- de projectverantwoordelijke of de instelling pleegt feiten die schade toebrengen aan het imago en de goede naam van de projectfinancier;
- er is een gegrond vermoeden van kwaad opzet voor het niet naleven van de bepalingen van de overeenkomst door de projectverantwoordelijke.

Artikel 11. Toepasselijk recht en bevoegde rechtbanken

Op deze overeenkomst is uitsluitend het Belgisch recht van toepassing.

Geschillen die uit deze overeenkomst voortvloeien en waar partijen niet tot een vergelijk kunnen komen, zullen uitsluitend ter beslechting van de Nederlandstalige kamers van de hoven en rechtbanken van Brussel worden voorgelegd

Artikel 12. Partiële nietigheid

De nietigheid of ongeldigheid van een clausule in deze overeenkomst heeft niet tot gevolg dat de overeenkomst in haar geheel nietig of ongeldig is.

Artikel 13. Volledige overeenkomst

Deze overeenkomst, met inbegrip van haar bijlagen die er integraal deel van uitmaken, is de enige die partijen bindt. Zij vervangt alle vorige overeenkomsten en afspraken.

Opgemaakt in twee exemplaren waarvan iedere partij verklaart een exemplaar te hebben ontvangen.

Gedaan te Brussel op ...24/01/17

Namens Projectfinancier
Marc Michils
Directeur Kom op tegen Kanker VZW

Projectverantwoordelijke
Professor dr. Gert De Meerleer

11 januari 2017

Voor de instelling
Professor dr. Marc Decramer

BIJLAGE 1: PROJECTDOCUMENTEN

1.1. PROJECTGEGEVENS

Titel project: Elective para-aortic radiation as part of combination therapy in prostate cancer patients with positive pelvic lymph nodes: a new step to improve clinical relapse free survival.

Projectverantwoordelijke: Professor dr. Gert De Meerleer

Vertegenwoordiger instelling: Professor dr. Marc Decramer

Instelling/Adres: Universitaire ziekenhuizen Leuven, Herestraat 49, 3000 Leuven

Ondernemingsnummer instelling: 419.052.173

Uitvoerders van het project: Gert De Meerleer, Steven Joniau, Wouter Everaerts

Toegekend bedrag: 269.304 euro

Looptijd projectondersteuning: van 15/11/2016 tot en met 14/11/2020

Deadlines indiening verslagen:

- **1° verslag:** 14/11/2017
- **2° verslag:** 14/11/2018
- **3° verslag:** 14/11/2019

Eindverslag: 14/02/2021

Betalingsplan:

De projecttoelage wordt in 48 maandelijkse schijven uitbetaald op

- rekeningnummer: BE78 3300 0057 5586
- van titularis: UZ Leuven
- met mededeling: PART studie – S59533 - KOTK

Bijkomende bepalingen:

! Indien de projectfinancier hierover nog niet beschikt dient een bewijs van de bankinstelling te worden toegevoegd waarin wordt bevestigd dat de instelling de titularis is van de vermelde rekening. Pas wanneer dit bewijs is overgemaakt aan de projectfinancier zal worden overgegaan tot betaling overeenkomstig de bepalingen uit onderhavige overeenkomst.
