# Supplementary material for: Benefits of Elective Para-Aortic Radiotherapy for pN1 Prostate Cancer Using Arc Therapy (Intensity-Modulated or Volumetric Modulated Arc Therapy): Protocol for a Nonrandomized Phase II Trial
Source: JMIR Res Protoc. 2018 Dec 13;7(12):e11256. doi: 10.2196/11256 (PMC6315267; doi:10.2196/11256)
Supplement: Multimedia Appendix 2 [file resprot_v7i12e11256_app2.pdf]

Kom op tegen Kanker
Koningsstraat 217 - 1210 Brussel
tel 02 227 69 69 - fax 02 223 22 00
info@komoptegenkanker.be

To whom it may concern,

The Board of Directors of Kom op tegen Kanker (Stand up to Cancer), the Flemish cancer society, has decided, on the advice of the biomedical committee, to support the project:

**'Elective para-aortic radiation as part of combination therapy in prostate cancer patients with positive pelvic lymph nodes: a new step to improve clinical relapse free survival'.**

Start project: 15/11/2016
Duration project: 48 months (14/11/2020)
Funding: 269.304 €
Principal Investigator: Professor Gert De Meerleer

Marc Michils

General Director Kom op tegen Kanker

komoptegenkanker.be
allesoverkanker.be
BTW BE 0442 528 054
IBAN BE03 4886 6666 6684
BIC KREDBEBB

Met de trouwe steun van: KBC, Eén en Radio 2
